# Assessment of the impact of quality improvement interventions on the quality of sick child care provided by Health Extension Workers in Ethiopia

**DOI:** 10.7189/jogh.06.020404

**Published:** 2016-12

**Authors:** Nathan P Miller, Agbessi Amouzou, Elizabeth Hazel, Hailemariam Legesse, Tedbabe Degefie, Mengistu Tafesse, Robert E Black, Jennifer Bryce

**Affiliations:** 1 Institute for International Programs, Department of International Health, Johns Hopkins Bloomberg School of Public Health, Baltimore, MD, USA; 2 United Nations Children’s Fund (UNICEF) Ethiopia Country Office, Addis Ababa, Ethiopia; 3 ABH Services, PLC, Addis Ababa, Ethiopia

## Abstract

**Background:**

Ethiopia has scaled up integrated community case management of childhood illness (iCCM), including several interventions to improve the performance of Health Extension Workers (HEWs). We assessed associations between interventions to improve iCCM quality of care and the observed quality of care among HEWs.

**Methods:**

We assessed iCCM implementation strength and quality of care provided by HEWs in Ethiopia. Multivariate logistic regression analyses were performed to assess associations between interventions to improve iCCM quality of care and correct management of iCCM illnesses.

**Findings:**

Children who were managed by an HEW who had attended a performance review and clinical mentoring meeting (PRCMM) had 8.3 (95% confidence interval (CI) 2.34–29.51) times the odds of being correctly managed, compared to children managed by an HEW who did not attend a PRCMM. Management by an HEW who received follow–up training also significantly increased the odds of correct management (odds ratio (OR) = 2.09, 95% CI 1.05–4.18). Supervision on iCCM (OR = 0.63, 95% CI 0.23–1.72) did not significantly affect the odds of receiving correct care.

**Conclusions:**

These results suggest PRCMM and follow–up training were effective interventions, while implementation of supportive supervision needs to be reviewed to improve impact.

Approximately 6.9 million children younger than five years of age died in 2011, with the vast majority of these deaths occurring in low– and middle–income countries [1]. Pneumonia, diarrhea, and malaria are among the leading causes of under–five mortality, with malnutrition as an important underlying cause [2]. Most under–five deaths can be prevented with available and cost–effective interventions [3]. Antibiotic treatment for pneumonia [4–6], oral rehydration salts and zinc for diarrhea [6–8], and artemisinin–based combination therapy for malaria [9,10] are effective interventions for preventing death from these diseases among children under five. However, poor and inequitable access to primary health care [11–13] and low quality of care [14–17] are important barriers. Integrated community case management of childhood illness (iCCM, see **Box 1** for definitions of key terms as applied in this study) is increasingly promoted as a key strategy to increase access to appropriate treatments for childhood illnesses, to reduce child mortality, and to improve equity in access to health services [18–20]. Through iCCM, community health workers (CHWs) are trained and equipped to manage common childhood illnesses (usually pneumonia, diarrhea, and malaria) in the community.

Box 1 **Definitions of key terms**

**Correct management:** This indicator of quality of care was used as the outcome variable in this analysis. Correct management is defined for this study as: The proportion of sick children for whom all HEW treatments matched gold standard treatments, including correct dose, duration, and frequency, and HEW referral matched the gold standard classification for referral for all major iCCM illnesses (pneumonia, diarrhea, malaria, malnutrition, and measles).

**Implementation strength:** This term refers to the quantity of effective program activities carried out to reduce child mortality. These activities include training, supportive supervision, and continued availability of essential iCCM commodities and supplies.

**Integrated community case management (iCCM):** In general, iCCM refers to the concurrent management of childhood pneumonia, diarrhea, and malaria in the community. iCCM in Ethiopia is integrated management by an HEW at the health post or community level of the following childhood illnesses: pneumonia, diarrhea, malaria, malnutrition, and measles.

**Quality of care:** For this study, quality of care refers to correct assessment, classification, treatment, and referral of patients according to iCCM clinical guidelines.

Since 2011, Ethiopia has been implementing iCCM, including treatment of uncomplicated pneumonia, diarrhea, malaria, malnutrition, and measles through Health Extension Workers (HEWs) in most regions of the country. All HEWs are literate women with at least a tenth–grade education, who receive a one–year pre–service training. The pre–service training included management of childhood diarrhea, malaria, and malnutrition; pneumonia was not part of the package prior to the introduction of iCCM. Following the training, they are recruited as government employees and deployed to work out of health posts at the kebele (sub–district) level. There are typically two HEWs working at one health post, which serves approximately 5000 people, the average population of a kebele. Through the iCCM program, HEWs treat children 2–59 months of age; sick children under two months are referred to a health facility [21].

The iCCM program was designed to strengthen the capacity of HEWs to assess, classify, and treat childhood pneumonia, diarrhea, malaria, malnutrition, and measles through training on iCCM, supportive supervision, provision of essential commodities, and enhanced monitoring and evaluation. Following the initial six–day clinical training on iCCM, a number of interventions were implemented within the iCCM program to ensure continued quality of care. These interventions were: follow–up training, performance review and clinical mentoring meetings (PRCMM), and supportive supervision. A description of the iCCM quality improvement interventions is provided in **Table 1**.

**Table 1:** Description of Ethiopia iCCM quality improvement activities

Activity	Description
Follow–up training	Refresher training in the health post within eight weeks of the iCCM training. This was a half–day to one–day visit by an iCCM trainer from the district or from an implementing partner agency. The purpose of the visit was to reinforce knowledge and skills learned during the initial iCCM training. They also carried out observation of sick child consultations, if possible, as well as reviewed sick child registers and administered case scenarios to assess HEW performance. Supervisors identified the HEW’s skills gaps and then focused on improving these during the visit.
Performance review and clinical mentoring meetings (PRCMM)	Two–day meeting held every six months (originally planned to be quarterly) at the *woreda* (district) level, during which approximately 20 HEWs met with supervisors, *woreda* health officials, health center staff. Zonal and regional focal persons sometimes also participated. *Woreda* iCCM facilitators and staff from implementing partner NGOs facilitated the meetings. On the first day, facilitators abstracted data from iCCM patient registers, reviewed registers with HEWs, and discussed issues related to quality of care and utilization of services. The second day was dedicated to clinical practice for HEWs in a health facility with feedback from facilitators. PRCMM guidelines are presented in Appendix S1 in **Online Supplementary Document(Online Supplementary Document)**.
Supportive supervision	Standardized supportive supervision on iCCM in the health post was performed on a quarterly basis. Supervisors were usually implementing partner NGO staff, and sometimes health center staff or *woreda* health officials. Supervisors used a standardized supervision checklist (Appendix S2 in **Online Supplementary Document(Online Supplementary Document)**), which included sections on case management, preventive services, supply of commodities, data collection, knowledge assessment, feedback, and next steps. Supervisors were instructed to either observe the HEWs conducting sick child consultations or review iCCM patient registers for completeness and consistency between recorded signs/symptoms, classification, and treatment.

HEW – health extension worker, iCCM – integrated community case management of childhood illness, PRCMM – performance review and clinical mentoring meeting

Ensuring quality of services provided through an iCCM program is essential to achieving mortality reductions. Assessments of IMCI and iCCM suggest that quality of care is variable and depends on the strength of program implementation, which is defined here as the level of delivery of key components of a program, such as training, supervision, and provision of commodities [22–29]. Program processes such as training and supportive supervision are presumed to lead to improved health worker performance, but, in practice, this is not always the case [30–35]. Most of the evidence available on the impact of program activities on quality of care pertains to health facility–based workers and is limited to formal training and supervision. A wide range of approaches to training and supervision activities have been implemented for iCCM [36], but little is known about the extent to which iCCM quality improvement activities are associated with the quality of care provided by CHWs. As part of an independent evaluation of the iCCM program in the Oromia Region of Ethiopia, we conducted a survey of health posts to assess iCCM program implementation strength and the quality of iCCM services provided by HEWs. These data provide an opportunity to assess associations between iCCM quality improvement interventions and quality of care provided by HEWs at health posts.

## METHODS

Data on iCCM implementation strength and quality of care were obtained through a cross–sectional survey of rural health posts in Jimma and West Hararghe Zones of Oromia Region, Ethiopia in which HEWs work. Oromia is the largest region in Ethiopia, with a population of approximately 30 million [37]. The survey was conducted in May and June 2012, about one year after initiation of iCCM implementation and during the low malaria season. The survey instruments were adapted from a survey of Health Surveillance Assistants in Malawi [26] and the World Health Organization (WHO) Health Facility Survey Tool [38].

We randomly selected 104 health posts from the 490 health posts that were implementing iCCM. To determine the sample size we assumed that proportions of key indicators were 50%, confidence level was 95%, non–response was 5% for health posts, 5% for HEWs, and 10% for patients, design effect to account for clustering of HEWs and patients within health posts was 1.3. Assuming an average of 1.5 HEWs and two children observed per health post, the sample sizes were expected to give estimates of health post– and HEW–level indicators with precision of +/– 10% percentage points and +/– 9 points for patient–level indicators.

Within health posts, all HEWs providing clinical services were included in the study. Children had to meet the following criteria to be included: 1) 2–59 months of age, 2) having at least one complaint consistent with an eligible iCCM illness, and 3) this was the initial consultation for the current illness episode. Eligible children were included in the study if they presented for care at the health post either spontaneously or because they were mobilized to seek care by the HEWs. If fewer than two children presented at the health post within one hour of the health post opening, the team supervisor, along with an HEW or community volunteer, recruited sick children from nearby households.

Data collectors were health professionals who had worked as iCCM trainers or supervisors. Survey personnel were trained for seven days, and all observers and re–examiners achieved at least 90% concordance with gold standard clinicians on three consecutive role play examinations.

During the survey, data collectors observed HEWs’ consultations with sick children and recorded details of the HEW’s assessment, classification, treatment, referral, and counseling. A separate data collector then carried out a re–examination of the same child to obtain the gold standard classification. Data collectors also conducted caretaker exit interviews, inspected commodities and patient registers, and interviewed HEWs. Data were entered directly into tablet computers using Open Data Kit (ODK) [39] as the data capture software and were stored in a Research Electronic Data Capture (REDCap) database [40]. Further details on the survey methods are available elsewhere [41].

Ethical approval was obtained from the Institutional Review Boards of the Oromia Regional Health Bureau and the Johns Hopkins University Bloomberg School of Public Health. Informed consent forms were read in local language to all participating HEW and caregivers of sick children and oral consent was obtained from all participants.

### Indicators

The primary indicators of implementation strength and quality of care were adapted from the WHO Health Facility Survey Tool [38] and the iCCM Global Indicators [42]. **Table 2** shows the list of indicators considered for use in this analysis. The outcome of interest was the proportion of children correctly managed for major iCCM illnesses (correct management), with a sick child as the unit of analysis. This is a summary indicator that includes management of pneumonia, diarrhea, malaria, malnutrition, and measles. A child was considered correctly managed if 1) the child received all recommended treatments and no unnecessary treatments; 2) the child was referred if and only if referral was indicated; and 3) the HEW prescribed the correct dose, duration, and frequency for all treatments.

**Table 2:** Selected indicators of iCCM quality of care and implementation strength and their proportions (or means (standard deviation), Jimma and West Hararghe Zones, Oromia Region, Ethiopia, 2012*

Variable	No.	% or mean	95% CI or SD
Outcome:			
Child correctly managed for iCCM illnesses	257	64.2	57.4, 70.5
Predictors:			
HEW received follow–up training within 8 weeks of iCCM training	134	46.3	36.7, 56.1
HEW attended performance review and clinical mentoring meeting	137	89.1	82.1, 93.5
Health post received at least one supervision on iCCM in the previous three months	100†	87.0	78.8, 92.9

CI – confidence interval, SD – standard deviation, HEW – health extension worker

*Indicators of quality of care and implementation strength were previously published by Miller et al [41].

†Three health posts excluded because HEWs reported not being present for majority of previous three months.

The predictors of interest were the training– and supervision–related interventions presented in **Table 1** that were intended to improve health worker performance (1. HEW received follow–up training within eight weeks of iCCM training, 2. HEW attended PRCMM, 3. Health post received at least one supervision on iCCM in the previous three months).

Child characteristics, such as age or severity of illness, can be important predictors of quality of care. However, because the objective of this analysis was to assess the relationships between the interventions to improve HEW performance and correct management, rather than to assess all possible predictors of correct management, we did not include child characteristics as predictors of interest in the analysis. The role of child characteristics as potential confounders is discussed below.

### Model selection and data analysis

Analyses for this manuscript were limited to HEWs who had received the standardized clinical training on iCCM. We assessed whether each of the three predictors of interest was associated with the outcome of correct management of childhood illness. Separate multivariate logistic regression models were developed for each predictor. For each of the three models (one for each predictor), we assessed potential confounders of the relationship between each predictor and the outcome. The potential confounders assessed were: zone, distance from the health post to the nearest referral health facility, availability of iCCM commodities, malaria risk of the health post catchment area, the number of sick child consultations at the health post in the previous month (caseload), HEW age, years of experience as an HEW, whether the HEW was from the kebele in which she worked, whether the HEW currently lived in the kebele in which she worked, whether the HEW intended to continue working as an HEW through the coming year (as a proxy for motivation), child sex, child age, whether the child had a severe illness, recruitment method that brought the child to the health post (spontaneous consultation, mobilized by the HEWs, or recruited by the survey team), and the other two predictors of interest.

We used purposeful selection of variables to assess potential confounders [43]. In the first step, we conducted bivariate logistic regressions of the outcome on potential confounding variables. Potential confounders with a *P*–value ≤0.25 from a Wald test were retained for further assessment. Starting with the variable with the weakest association, these variables were assessed as confounders for each of the models. We then compared a full model, with the predictor and all of the variables retained in step one above, to a null model with one of the potential confounders removed. If removing the variable changed the coefficient of the predictor of interest by ≥15%, the variable was included in the final model as a confounder. This was repeated for each of the potential confounders.

Finally, we performed multivariate logistic regression analyses using two–level random intercept models with child and health post levels to assess associations between each of the quality improvement interventions and the outcome, controlling for the retained confounders. An alpha level of 0.05 was used for tests of statistical significance. All analyses were carried out in Stata 13 [44].

## RESULTS

All but one of the selected 104 health posts implementing iCCM were successfully surveyed, giving a final sample of 103 health posts. All 137 HEWs encountered in health posts and 257 sick children were included in the survey. Sample characteristics and detailed results on iCCM implementation strength and quality of care are available elsewhere [41].

Bivariate analyses showed that PRCMM (OR = 6.45, 95% CI 2.22–18.73) and follow–up training (OR = 2.25, 95% CI 1.24–4.07) were significantly associated with the outcome. Supervision on iCCM (OR = 1.15, 95% CI 0.44–2.99) was not significantly associated with correct management.

The final models for each predictor and the results of the multivariate logistic regressions are shown in **Table 3**. Attendance at PRCMM had the largest association with correct management, controlling for other covariates. Children who were managed by an HEW who had attended a PRCMM had about eight times the odds of being correctly managed (OR = 8.3, 95% CI 2.34–29.51), compared to children managed by an HEW who had not attended a PRCMM. The HEW receiving follow–up training also significantly increased the odds of the child receiving correct management (OR = 2.09, 95% CI 1.05–4.18). The HEW receiving supervision on iCCM (OR = 0.63, 95% CI 0.23–1.72) was not significantly associated with a child receiving correct care.

**Table 3:** Final models and results of multivariate analyses of associations between iCCM quality improvement interventions and correct management of major iCCM illnesses, controlling for selected covariates, in children 2–59 months, Jimma and West Hararghe Zones, Oromia Region, Ethiopia, 2012

Predictor variable	Covariates	Number. of children (N = 257)	% children correctly treated	OR (95% CI)	*P*–value
Child managed by HEW who attended PRCMM:	–				
Yes		233	68.2	8.3 (2.34, 29.51)	<0.001
No		24	25.0	Ref.	
Child managed by HEW who received follow–up training within 8 weeks of iCCM training:	Child with severe illness				
Yes		116	74.1	2.09 (1.05, 4.18)	0.037
No		141	56.0	Ref.	
Child managed in health post that received at least one supervision on iCCM in the previous three months	PRCMM; follow–up training; child with severe illness				
Yes		225	65.3	0.63 (0.23, 1.72)	0.369
No		29	62.1	Ref.	

OR – odds ratio, CI – confidence interval, HEW – health extension worker, iCCM – integrated community case management of childhood illness. PRCMM – performance review and clinical mentoring meeting

## DISCUSSION

Previous studies that looked at associations between program inputs and health worker quality of care in low–income countries have typically focused on training and supervision of health workers in health facilities. These studies of health facility workers have had mixed results [30,45], with some showing significant or near–significant associations between indicators of quality of care and training [46–52] and others showing little or no association [32–34]. Similarly, supervision has been shown to improve health worker performance in some studies [53] and to have no or little effect in others [33,34,54,55]. Reviews of interventions to improve health worker performance found that quality improvement interventions with multifaceted interventions, such as training plus supervision, generally have a greater effect and that supervision with feedback is usually beneficial [30,45].

Only a few studies have assessed the effect of quality improvement interventions on quality of care provided by CHWs. The evidence from this small number of studies is also mixed. Some found positive associations between quality of care and training [55,56] and supervision [56], while others showed no significant associations with training [57,58] or supervision [58]. Two recent studies in Ethiopia showed associations between supportive supervision [59] and PRCMM [60] and improved consistency in iCCM patient registers between HEWs’ assessment, classification, and treatment of sick children.

We found that HEW attendance at a PRCMM and follow–up training were both significantly associated with the outcome. PRCMM, in particular, had a very strong association with correct management. These are important findings because these interventions are not always included in quality improvement programs.

These findings should be interpreted with caution, as there is the potential for unmeasured confounding. It is possible that the HEWs who provided lower quality care were less motivated and did not attend a PRCMM. This would spuriously inflate the observed association between PRCMM and the outcome. During the survey, a small number of HEWs (16) reported that they did not plan to continue working as an HEW through the coming year. Because this variable may be related to HEW motivation, we assessed whether there may be a negative association between attending a PRCMM and intention to continue as an HEW. In fact, all of the HEWs who reported an intention to leave their jobs as HEWs had attended a PRCMM. This is far from an ideal proxy for motivation and a very small sample size, but it does somewhat weaken the argument that HEW motivation confounded the relationship between correct management and PRCMM.

Routine supervision for iCCM was not associated with the outcome. PRCMM and follow–up training may have been more effective than routine iCCM supervision because they were longer and more intensive, and were more specifically focused on reviewing case management guidelines and clinical practices. On the other hand, the observed lack of association between supervision and correct management may reflect a lack of focused improvement of clinical skills, with more emphasis on data collection and register review. The effect of supervision may also have been reduced in a context where a large majority of HEWs had received PRCMM. Supervision may have had a limited *additional* effect after the strong effect of PRCMM.

The discrepancy between these results and the previous study showing a significant association between supervision and quality of care among HEWs [59] is likely because of the use of different outcome variables. Ameha et al. used consistency between classification and treatment from patient registers as the outcome variable. We have previously shown that register review overestimates quality of care compared to estimates derived from observation of consultations and re–examination of sick children [61]. Because supervisions focus on register review, it is likely that they improve recording of cases, but this may neglect improvement of assessment skills, which can lead to incorrect classification.

Further research is needed on how to improve the quality and effectiveness of training and supervision interventions to achieve maximum impact. For example, supportive supervision may be more effective if it includes a component of case observation with feedback, rather than relying entirely on register review. However, the feasibility of doing this in a setting of small numbers of consultations needs to be assessed.

This study has several limitations. First, the observational design of the study limits the conclusions about causality that can be drawn. The absence of a comparison group makes it impossible to rule out unmeasured variables that may have confounded the observed associations between the interventions and correct management. Second, health workers may have performed better under observation than they would under normal circumstances [62,63]. Third, data on the predictors were based on HEW recall, which may be biased toward having received the interventions, although Hazel et al. found that HSAs in Malawi accurately reported implementation strength indicators [64]. Finally, because HEWs receive relatively more training and compensation than many CHW cadres in other countries, these results may not be generalizable to other contexts where iCCM is implemented.

The results of this study, as well as large variations in impact from quality improvement interventions studied in other contexts, suggest that it may be the quality of implementation of interventions, more than their inherent characteristics, which determines their effectiveness. Quality improvement interventions for health workers can lead to improvements in quality of care, but they do not necessarily do so. Therefore, efforts should be focused not only on achieving high coverage of quality improvement interventions, but also on the content and quality of these interventions to ensure that they have the expected impact. The positive associations between follow–up training and especially PRCMM suggest that these interventions should be considered as complements to standard trainings and supportive supervision to improve the quality of iCCM services.
