# Supplementary material for: Assessment of the impact of quality improvement interventions on the quality of sick child care provided by Health Extension Workers in Ethiopia
Source: J Glob Health. 2016 Aug 19;6(2):020404. doi: 10.7189/jogh.06.020404 (PMC5012232; doi:10.7189/jogh.06.020404)
Supplement: Online Supplementary Document [file jogh-06-020404-s001.pdf]

Online Supplementary Document
Miller et al. Assessment of the impact of quality improvement interventions on the quality of sick child care provided by Health Extension Workers in Ethiopia
J Glob Health 2016;6:020404

Appendix S1

UNICEF

Ethiopia Integrated Community Case Management of Common Childhood Illnesses Performance Review and Clinical Mentoring Meeting Guide

Working Draft

Contents

Table 1. Activity Checklist 3

Table 2. Agenda 4

SECTION 2: INTRODUCTION 8

SECTION 3: REGISTER REVIEW 9

SECTION 4.1 CLINICAL PRACTICE 13

ANNEXES

Annex 1: Flip Chart Rationale, objectives and major activities 15

Annex 2 Strategies to increase utilization of iCCM service 16

Annex 3 Newborn Health 17

Annex 4: Case scenarios for register filling exercise 18

Annex 5 Form C, ... 20

Annex 6 Record Forms........ **Error! Bookmark not defined.**

Annex 7 SC and SYI compilation Forms 30

Annex 8 Sample Planning Template for health post 31

Annex 9 Sample Planning Template for Woreda health Office........ 33

Table1. Activity Checklist

	Type of Activity	Time
	Day One	
	Registration	
**SECTION 2**	**Introduction**	
**Activity 2.1**	Introduction of participants	10 min
**Activity 2.2**	Lying ground rules of the meeting	5
**Activity 2.3**	Presenting the agenda of the meeting	5
**Activity 2.4**	Presentation of the objectives–***Flip chart 1***	15 min
**SECTION 3**		
**Activity 3.1**	Group work: Review of iCCM registration books for *completeness* and *consistency*	180 min
**Activity 3.2**	Plenary discussion: group work presentation and discussion	30
**Activity 3.3**	Present the summary of previous iCCM follow up visits findings to health posts/HEW (analysis of form C) focusing on the implementation strengths and challenges- ***Flip chart-2***	20 min
**Activity 3.4**	Discussion and summarization of strengths, weaknesses, challenges and solutions; *with special emphasis on service utilizations-bottlenecks and solutions* ***–Flip chart-3***	60 min
**Activity 3.5**	Exercise on Case scenarios 1, 2, 3 and 4 with feedback at the end (Optional)	60 min
	Activity 4.3 to 4.5 will be covered on day1 when a 2nd day (clinical practice) is not added	
	**Day two (**in the first round of iCCM PRMM**)**	
**Section 4**	**Clinical practice**	
**Activity 4.1**	Assign cough or difficult breathing, diarrhea, fever and SAM to HEWs	180
**Activity 4.2**	Feedback of the clinical practice	10
**Activity 4.3**	Planning a 3 month plan	60
**Activity 4.4**	**Closing**	
**Activity 4.5**	Distribution of supplies when as needed	

Table2. Agenda

Time	DAY ONE: SUNDAY (Sunday, if this is the first round for the HEWs)	Responsible Facilitator
	**Activity**	
8:30-8:40	**Activity 2.1:** Registration	
8:40-9:00	**Activity 2.2 ; 2.3 and 2.4 :** Introduction of participants , lying ground rules, and presenting the agenda	
9:00-9:30	**Activity 2.5:** Presentation of the objectives, rationale and expected outcomes of the PRM/mentoring –*Flip chart 1*	
9:30-9:45	**TEA BREAK**	
9: 45-12:30	**Activity 3.1**: Group work- review of iCCM registration books from each health posts for case load; completeness and consistency –using form-C modified for PRM and abstraction forms and ; give feed back	
12:30-2:00	**LUNCH BREAK**	
2:00-2:30	**Activity 3.2:** Plenary discussion: group work presentation and discussion	
2:30-2:50	**Activity 3.3:** Presentation of summary of previous iCCM follow up visits findings to health posts/HEW (analysis of form C) focusing on the implementation strengths and challenges – *Flip Chart 2*	
2:50-3:50	**Activity 3.4:** Discussion and summarization of strengths, weaknesses, challenges and solutions; with special emphasis on service utilizations-bottlenecks and solutions-Annex 3	
3:50-4:05	**TEA BREAK**	
	**Activity 3.5:** Exercise on case scenarios 1, 2, 3 and 4 with feedback (*Optional*)	
4:30-5:30	**Activity 3.6: for subsequent one day review meetings**<br>— Develop the next quarter plan,<br>— Distribution of supplies<br>— Closing	
**DAY TWO: MONDAY**	**(first round** performance review and mentoring meeting **PRMM)**	
	**CLINICAL PRACTICE**	
8:30-11:30	**Activity 4.1**<br>— Assign cough or difficult breathing, diarrhea, fever and SAM to HEWs<br>— Observe and mentor each HEW	
11:30-12:30	**Activity 4.1:** Introduction to the routine health post reporting -reaching common understanding	
12:30-2:00	**LUNCH BREAK**	
2:00-3:00	**Activity 4.1:** Develop a three month plan and agree on the way forward	
	**CLOSING**	
	Distribution of supplies as needed	

SECTION 1: PREPARATION

Planning and organizing an iCCM performance review and mentoring meeting /PRMM/

A productive and effective PRMM needs good planning and preparation ahead of time. In planning for PRMM, the coordinator needs to answer the following questions:

1.1 Who needs to be included?

Health extension workers (HEW) who has received iCCM training and is working in a health post for at least three months will be eligible. In addition HEW supervisor, primary health care unit /PHCU/ director, woreda HEP focal person, and the Woreda health office head and if time allows zonal and regional focal persons can participate. The iCCM implementing partner will involve its project officers and facilitate the whole process

1.2 How many participants will be in the PRMM?

This PRMM, that aims to improve the technical skill and knowledge of HEW and supervisors, should accommodate a number, which is adequately handy. The number should not exceed 24 HEW (preferably 20) participants per meeting.

1.3 Who is responsible for what?

A PRMM needs a coordinator and facilitators to plan, organize and manage the process. Facilitators need to divide the tasks among themselves before hand and be well prepared on their respective responsibility. The coordinator organizes the meeting and coordinates activities while the facilitators provide information, organize tasks, supervise skill practice, evaluate the progress of participants and provide feedback.

1.4 How many facilitators are needed and what qualifications should they have?

One facilitator to three health posts (6-8 HEW) will be needed for the meeting. The facilitator should be a person who has been trained in iCCM TOT/supervisory skill (the seven days course) and be able to properly review iCCM registers and give feedback to HEWs. In the same line he/she should be familiar to the PRMM guide. There should be a preparatory meeting to become conversant with and coach persons who will participate as facilitators

1.5 How long should the PRMM last?

This PRMM needs two full days during the first round and then one day during the subsequent rounds

1.6 What facilities are needed?

a) Invitation

- Check with the relevant people that there are no other competing tasks on the same date and identify a date that works
- Using invitation letter or equivalent means of communication from the woreda invite participants well ahead of time. It is also crucial to check and follow through telephone or any other means of communication to make sure that the message has reached the intended people or institutions.

b) Venue

A large room with chairs and tables is required in which all participants can sit comfortably. Space is also needed for small group exercises/discussions and demonstrations.

c) Clinical setting selection and arrangement for iCCM PRMM

The PRMM will include clinical practice particularly during the initial rounds; the training center should be near a health facility to which the participants have access. The health center should have adequate number of sick children for the HEW to practice on and acquire the desired case management skills. The course coordinator officially communicate the health facility is. It is necessary to assign a focal person who selects and keeps sick children for the participants according to a given schedule. Using communities for clinical practice is another option.

d) Logistics needed for the iCCM PRMM

Ensure you have all the materials before the beginning of the PRMM. Use the checklist below

HEWs should come with both their registers that has been filled with case management information, chart booklets, exercise booklets, wrist watch, MUAC tape

Table 3. Logistics Checklist

S.N	Tasks or materials	Qty	Responsible
1.	Registration form	1	organizer
2.	Form C	1 for each facilitator	organizer
3.	Compilation forms for sick young infant and sick child for facilitators	1 for each health post	
4.	Flip chart	1	organizer
5.	Pre-prepared flip chart- presentation on objectives, expected outcomes,	1	organizer
6.	Chart booklet	1 for each participant	Participant
7.	Copy of A3 size registers(SC and SYI)	1 for each facilitator	organizer
8.	iCCM Facilitator	1 for each facilitator	facilitator
9.	RM Guide	1 for each facilitator	organizer
10.	iCCM Registers	2 from each health post	HEWs
11.	MUAC tape, FHC	1 for each HEW	HEW
12.	All HEWs from 5-10 HP (max of 24 HEWs)	20-24	organizer
13.	Zonal iCCM focal person-important in the initial meetings	1	organizer
14.	Woreda focal person-important	1	organizer
15.	Woreda Health Office Head(at least for the first session)	1	organizer
16.	Health center director-mandatory	1	organizer
17.	HEW supervisor-mandatory	1	organizer
18.	Performance Review meeting(PRM)/mentoring agenda	1 for each participant	organizer
19.	Facilitators to participant ratio	1 to 3 Health post (6-8 HEWs)	organizer
20.	Compiled and summarized results from previous follow-up visits	Flip chart	organizer
21.	Case scenarios 1, 2, 3 and 4 with answers (optional-done when needed)	1 for each participant	organizer
22.	Stationary –not book, pencil and sharpener	1 for each participant	organizer
23.	Supplies/logistics	as needed	organizer
24.	Planning format	1 for each participant	organizer
25.	Routine health post Reporting format	1 for each participant	organizer
26.	Certificate(for those who did not receive it		

SECTION 2: INTRODUCTION

Activity 2.1 Registration of participants

Using a standard form prepared for this meeting register all participants

Activity 2.2 Introduction of participants

Introduce yourself and participants in a simple and entertaining way covering the following points: Use a pre-prepared flip chart. (*Flip chart*)

- Name
- Working place
- Responsibilities
- Your favorite animal and why
- Expectation from the review and mentoring meeting

One of the facilitators writes the expectations of the participants on a flip chart, so that it can be compared with the review and mentoring objectives.

Activity 2.3 Laying ground rules and announces any administrative arrangements

- Discuss with participants and get agreement.
- Discuss with participants to lay ground rules to follow during the review and mentoring meeting time. Write it on a flip chart and paste it on visible site (Flip chart)
- Agree on:
  - Starting time, finishing time, tea/lunch break time
  - Punctuality, active participation, no side-talks etc.
- Explain if there are any administrative arrangements (tea, coffee and lunch breaks)

Activity 2.4 presentations of the meeting agenda

Present the agenda of the meeting briefly

Activity 2.5 Presentation of the objectives of the performance review and mentoring meeting
(Flip chart 1 in annex 1)

- Using a flip chart prepared ahead of time present the objectives and rationale of the performance review and mentoring meeting.
- Ask participants if they have any question or comment on what you have presented

SECTION 3: REGISTER REVIEW

Activity 3.1 Review iCCM registers for completeness, consistency and case load (coverage); record findings and give feed back

- Form 3 - 4 groups of health posts with their HEWs (6-8 HEW). Assign one experienced facilitator who can review the register for completeness and consistency and give feed back
- Explain to HEW that this session is a continuation of the training and they should not take it as an exam or evaluation; encourage them to be ready to ask, comment and learn more,
- Explain and demonstrate what completeness and consistency means
- Facilitators will complete the compilation forms(SYI and SC)
- Facilitator reviews the registers of SC and SYI-use form C for each HP as a guide and for recording. After the first one; ask the next HEW to briefly describe what is in her register
- Encourage the HEWs to refer to the chart booklet for all iCCM tasks
- Record information on form C-selected two recent classification for each health post
- Give feedback –start from the strengths and then the weaknesses
- Sign the date of the review on the register-at the end of the last case as this will allow starting place for the next review meeting or supervision

Note for the facilitator

- Do the above activities health post by health post until all the health posts are finished; while HEWs from a specific health post get their registers reviewed and received feedback the other HEWs will observe the process until their turn comes and until the activity 3.1 is over

- The subsequent review meetings could be more participatory than the first one by exchanging registration books among HPs and allowing discussion

- The subsequent review meetings will start on reviewing previous plan performance

- Understand how to fill the compilation form before the exercise

- During the register review discuss experience in iCCM implementation including successes and challenges and let one HEW to be a note taker and present it to the plenary during activity 3.2

Activity 3.2 Plenary discussion on HEWs experience on iCCM implementation

- After the register review; ask note taker HEWs to share experience on iCCM implementation, strengths, challenges and solutions
- Encourage active participation
- when all participants finish their comments; add the information identified by facilitators from activity 3.1 using summary of form C

Activity 3.3 Present the summary of previous iCCM follow up visits findings to health posts/HEW

- Present a summary of findings from previous iCCM follow up and supervisory visits of health posts in the same area where HEWs have come from, prepared ahead of time
- Focus on the strengths and success stories(case history)-if any, and challenges in iCCM implementation
- Ask participant for comments or questions
- Summarize the discussion

Activity 3.4: Service utilizations-bottlenecks and solutions

- Describe iCCM service utilization and its current status. Give the following example on expected cases and coverage:
  - In a kebele with a total population of 5,000
  - U5 700 (13.5% x 5,000)
  - Expected pneumonia cases: 700 x .3 =210
  - Assuming equal monthly distribution or ignoring seasonal variation there will be about 18 cases per month(210 ÷12)
- Ask one or two volunteer HEW to describe pneumonia case load so far, ask the total population of their respective kebeles and calculate expected pneumonia cases per month show the gaps
- Brainstorm on the possible bottlenecks of low utilization for both the SYI and let another facilitator writes of a flip chart
- Brainstorm on the possible bottlenecks of low utilization for both the SC and let another facilitator writes of a flip chart
- Brainstorm on the strategies overcome the above listed bottlenecks for low utilization to achieve acceptable coverage
- Present strategies to increase utilization of iCCM service (annex 2), summarize the discussion and reach consensus. (keep the summary points they will be utilized during the planning session for the next quarter towards the end of the meeting

Note for the facilitator

Present annex 3 on a flip chart and describe the magnitude of newborn health issues and reinforce the role of HEP in reducing current level of newborn deaths

Emphasize the fact the there are no cases of sick young infants is because of lack of care seeking-not because morbidity is low in this age group. The reverse is true and due to deeply rooted cultural beliefs mother and newborns are confined for about 45 days and don't seek care outside their homes.

For subsequent review meeting discussion may include other areas depending on the prevailing bottleneck

Activity 3.5: Exercise on Case scenarios 1, 2, 3 and 4 (Optional)

- It is very important HEWs are able to properly fill the registers which is one aspect of the quality of case management they provide- if you are convinced that more than a 4th of HEW still lacks the knowledge to properly fill registers (from activity 3.1-register review) let participants do exercise on the case scenarios taken from the iCCM facilitators guide page 103 activity 11.4, Amharic (*annex 3 in this guide*)

- Tell HEWs from the same health post to be together and turn on an empty page from the last pages of both their registers where they are going to record the case scenarios as you read for them

- Read the case scenarios loudly and make sure all of them record all the information you give

- After you have read the case and made sure they have recorded the information tell them to write the correct classifications, treatments and shortest correct follow up date (if the sick child or young infant needs referral tell them to write the correct information in the appropriate space)- give them enough time to do the task for each case scenario

- Remind them to use their chart booklet for case management and FHC for counseling- start by asking them what job aid they should use to provide correct case management

- As one facilitator reads the case scenarios and HEW fill the registers; the other facilitators observe the HEW how they are doing the task

- Give feedback: make sure all HEW have completed the task

DAY TWO

SECTION 4.1 CLINICAL PRACTICE

Following the initial training course taking HEWs to a health facility and allowing them practice case management under your observation will create more opportunity to improve their skills and this could be the only opportunity to conduct observation of case management-which is crucial to assess the HEW skill in case management . At the same time it gives you a snap shot of areas where the HEW needs support and mentoring.

- Review the chart booklet before going to the clinical session
- Divide participants in two small groups based on the available health facilities then divide the two groups further into small groups of two to four participants based on available cases o practice on patients
- Distributed copy of A3 register(SC and SYI)
- Assign experienced facilitators for each small groups
- Initially an experienced facilitator should demonstrate the correct management tasks-this time all the case management tasks are to be done (like the last day of iCCM training)
- Select cases with cough or difficult breathing, diarrhea, fever and very severe disease/possible serious bacterial infection in the young infant
- Distribute the cases among the participants and let them complete the management tasks. Observe and support as needed. Reinforce weak tasks like counseling, using FHC and demonstration giving of first dose. Ensure the children receive the proper care from the health facility staff
- Focus on the weak HEWs but not leave out the others
- Provide feedback for each HEW and or each small group based on their clinical performance
- At the end summarize tasks, thank the health facility staff and depart

Activity 4.2 Feedback on clinical practice

- Briefly ask participants to reflect on the exposure of clinical practice in the morning
- Present a brief summary of the clinical practice to plenary
- Emphasize the need for use of chart booklet at all time, need for being familiar to different section of the chart booklet, counseling, about oral drugs, newborn care and use of service by the sick young infant

Activity 4.3 Develop and agree on next three month plan

- Form two groups:
  - HEW according to their respective health posts
  - Woreda health office head, focal person, PHCU directors and HEW supervisors
- Distribute two copies of planning form and assist participants to prepare a plan for their specific health posts, health centers and woreda health office – the plan will be based on the identified strengths, challenges and solutions during the register reviews, and discussions
- While a copy remain with the HEW and PHCU, one will be submitted to the PHCU/woreda/partner
- This activity will be done during every PRMM

Activity 4.4 highlighting the way forward and closing

- The PHCU/woreda person will highlight the way forward and close the meeting

Activity 4.5 logistic distribution

- Do distribution of supplies, equipment or job aids as needed

ANNEXES

Annex 1: Flip Chart Rationale, objectives and major activities

Introduction:<br>• Training is only an entry point to the implementation of a program<br>• For any program to be successful, giving quality training alone will not be enough; conducting follow up and supportive supervision will be of critical importance<br>• ICCM Start up follow up for trained HEW is needed 4-6weeks after training followed by regular supportive supervision<br>• In addition regular performance review meeting with mentoring for a group of HEW will be needed	Objectives:<br>1. Reinforce integrated case management skills and assist HEWs to transfer these skills to actual practice;<br>2. Identify problems faced by HEWs in managing sick children and help solve these problems;<br>3. Gather information on the performance of HEWs, and explore conditions that influence performance in order to improve the implementation of iCCM of sick children under five in the future.<br>4. Distribute supplies when needed
Expected outcomes<br>At the end of this meeting:<br>• HEW's case management skills will be reinforced<br>• HEWs will be able to fill iCCM registers more correctly<br>• iCCM information collected from registers and other sources<br>• Strengths and weaknesses in iCCM implementation identified<br>• Solutions to weaknesses identified and agreed<br>• Way for ward agreed and next quarter plan developed	Summary of major activities<br>• iCCM registers from each health post will be reviewed for consistency and completeness<br>• Feed back and mentoring given to HEW<br>• Case management information collected from registers using standard form<br>• Summary of previous iCCM follow up/supportive supervision findings will be presented

Annex 2 Strategies to increase utilization of iCCM service

Under the mandate of HEWs	PHCU director, woreda. Zone, RHB and iCCM partners
• Identify barriers to service utilization and act on them<br>• Give a quality service (be competent, committed and friendly to care takers)<br>• Make the health post ready for service and communicate the days and hours service is available (open for some time every day)<br>• During every home visits ask if there is any sick under five and manage –use the record form and fill in the register when you come back to the HP<br>• Create demand (sensitize the community using different gatherings, plan and review with VCHW and community leaders)<br>• Proper management of drugs, supplies, equipment<br>• Monitor the level of service utilization against expected case load –analyze and use data for action	• Use the HMIS data on cases of iCCM conditions treated at the HC and HP level to asses utilization<br>• Have an action plan that includes increased utilization as a target at both health posts and health centers- and implement and support all the strategies<br>• Make sure quality case management at both health centers is provided and referral link is improved<br>• Regular Supportive supervision and follow up and performance review meetings<br>• Ensure the availability of essential supplies, job aids and equipments<br>• Follow up if HP not opened regularly and both HEW are away<br>• Regular collection, analysis and use of data from health post and HC for decision

Annex 3 Newborn Health

Status and HEP's role in reducing neonatal mortality

- 120,000 newborns die annually in Ethiopia (EDHS 2005)
- In Ethiopia newborn death constitutes about 40% under five deaths-this is huge risk given that fact newborn period is very short period in child's life
- Two third of newborn death occurs in the first week and from these deaths two third occur on the first day
- 94% deliver at home - lack of intrapartum and postnatal care and no increase in coverage of skilled care
- MDG- 4 can only be achieved if neonatal deaths are addressed
- Approx 33% of neonatal deaths could be averted with outreach and family or community-based interventions, feasible now thru HEW
  - Make childbirth wanted and safe
  - Family planning, antenatal care, clean and safe delivery
  - Give newborn babies a healthy start
  - Immediate newborn care, post natal care through two PNC home visit with in the first seven days after delivery, promotion of breastfeeding,
  - Prevent infections
  - Treated nets for malaria, nutrition, immunization, hygiene and Prevention of Mother to Child Transmission of HIV
  - Manage infections
  - Treat childhood infections, identify and refer promptly newborn and maternal infections

Annex 4: Case scenarios for register filling exercise (Optional)

Sick young infants

Case 1: Young infant ***Ababu*** *is a* ***3-week****-old infant from* ***sub-kebele 2***, ***Waka kebele*** *and* ***Zana woreda.*** *He has a* ***weight of 3.6 kg*** *and axillary* ***temperature of 36.5 °C***. *He is brought to the clinic because he is having* ***difficult-breathing***. *The health extension worker first checks the young infant for signs of possible bacterial infection. His mother says that Ababu has not had convulsions, has no feeding problem. The health extension worker counts* ***74 breaths per minute***. *On a repeat count she counted* ***70 breaths per minute***. *She finds that Ababu has mild chest indrawing. He has no grunting. The umbilicus is normal, and there are no skin pustules. Ababu is calm and awake, and his movements are normal. He does not have diarrhoea or jaundice. His mother was tested for* ***HIV*** *to be* ***negative*** *two months back. Ababu has taken* ***OPV-0 and BCG*** *at birth.*

Case 2*: Young infant* ***Hanna is 7 weeks*** *old from* ***Sub-kebele 1, Shasha kebele*** *and* ***Dara woreda***. *Her* ***weight is 3 kg***. *Her axillary temperature is* ***36.5°C***. *Her mother has brought her because she has* ***diarrhoea*** *with* ***blood in the stool*** *for* ***3 days***. *The health extension worker first assesses her for signs of severe disease or bacterial infection. Her mother says that she has not had convulsions. Her breathing rate is* ***58 per minute***. *She was sleeping in her mother's arms but awoke when her mother un-wrapped her and moving her arms and legs normally. She has slight chest indrawing, no grunting. Her umbilicus is not red or draining pus. She has a rash in the area of her diaper, but there are no pustules.*

When the health extension worker asks the mother about Hanna's diarrhoea, the mother replies that it began 3 days ago, and there is blood in the stool. Hanna is ***crying***. *She stopped once when her mother put her to the breast. She began crying again when she stopped breastfeeding. Her eyes look not sunken. When the skin of her abdomen is pinched, it* ***goes back slowly***.

Further assessment showed that Hana's mother was tested for ***HIV*** *to be* ***negative*** *three months back. Hana is* ***not immunized*** *yet.*

Record the young infant's signs; classify and write the correct treatment on the registration book.

Sick children

Case 1: Leya*, from* ***sub-kebele Rawa, kebele Dola*** *and* ***Titi*** *woreda is* ***5 months old*** *infant. She* ***weighs 5 kg***. *Her temperature is* ***36.5°C***. *Her family brought her to the clinic because she* ***feels hot*** *and has had* ***cough for 2 days***.

She is able to drink. She has not vomited or had convulsions, and is not convulsing, not lethargic or unconscious.

The health extension worker said, "I am going to check her cough now." She counted ***43 breaths*** *per minute. There was no chest indrawing and no stridor when Leya was calm. Leya did not have diarrhoea.*

*"Now, I will check her fever," said the health extension worker. Leya lives in an area where many cases of malaria occur all year long (**high malaria risk**). Her mother said, "Leya has felt hot on and off for 2 days." She does not have stiff neck or runny nose.*

*Leya has a **generalized rash**. Her **eyes are red**. She has **mouth ulcers**. They are **not deep** and extensive. She does not have pus draining from the eye. She does not have clouding of the cornea.*

The HEW did RDT on Leya which turned out to be positive for P. falciparum.

*Leya has MUAC of 13 cm, and no **oedema** of both feet. She has **no palmar pallor***

She and her mother are not tested for HIV** any time in the past. Leya **has taken BCG, OPV-3, Pentavalent-3 and Pneumococcal-3

Record the child's signs and classify them on the registration book.

Case 2: ***Meron is 18 months old** she comes from **sub-kebele 4, kebele Selam,** and **Mar woreda**. She **weighs 7 kg**. Her temperature is **38.5℃**. Her mother brought her today because the child has **felt hot** and **has a rash.***

*The health extension worker checked for general danger signs. Meron was **not able to drink**, has not vomited, has not had convulsions, and was not convulsing during the consultation. She was not lethargic or unconscious. She does not have cough or difficult breathing. She does not have diarrhoea.*

*Because Meron's mother said the child felt hot, and because her temperature is 38.5℃, the health extension worker assessed her for fever. Meron lives where there is a **high malaria risk**. She has had **fever for 5 days**. Her **rash is generalized**, and **she has red eyes**. She does not have a stiff neck. She does not have a runny nose. The HEW has done **RDT which was negative for malaria.***

*The health extension worker assessed her for signs of measles complications. Meron **does not have mouth ulcers**. There is **no pus draining from the eye** and **no clouding of the cornea**. Meron does not have an ear problem.*

*The health extension worker next checked her for malnutrition or anemia. Meron has a **MUAC of 10 cm.** She **has refused to eat or drink anything**. There is **no palmar pallor**. She **does not have oedema of both feet.***

*She and her mother **are not tested for HIV** any time in the past. The health extension worker has confirmed from the immunization card that Meron has **completed her immunization** at age 9 months*

*She **has received vitamin A and Mebendazole 4 months back***

Record the child's signs; classify and write the treatment on the appropriate registration book.

- Answers to the case scenarios (to be followed)

Annex 5 Form C,

Form C: Program Review/mentoring meeting (PRMM)

Identification

1.1	Date of visit (dd /mm/yyyy)
1.2	How many weeks ago was the last visit: ___weeks.
1.3	Region: __________ Zone: __________Woreda: __________Kebele/H. Post: __________
1.4	Kebele's total population; __________; Total U5 children in the kebele__________
1.5	Name of supervising health centre: __________
1.6	Name of HEW in charge__________
1.7	Lead supervisor's name: ___________Responsibility (e.g. HEP supervisor, iCCM coordinator, HEP focal person) __________Organization (e.g. MOH, Implementing Partner [specify name__________
1.8	Was Direct Case Observation made? Yes__ No ___Total number of sick U5 observed: ____;<br>Number of Sick Children (2 -59 months)___ Number of Sick Young Infant (0 up to 2 months)__<br>(*if you get sick children during your visit do direct case observation using the appropriate recording form annexed at the end of this form*)
1.9	ICCM registration book reviewed: Yes __________No __.Total number of sick U5 children reviewed __________; Number of Sick Children (2 -59 months ______, Number of Sick Young Infant (0 up to 2 months) ____
1.10	Total number of HEWs in Health Post: 2__Name of HEW supervised: 1. ___________; 2) __________; 3)______________________
1.11	How many Voluntary Community Health Workers in this kebele? __________<br>a. Which of the below activities do they perform:<br>i. Mobilizing families to seek iCCM services ______Yes ___ No ___ not yet begun<br>ii. Health promotion activities ___________Yes ___ No ___Not yet begun

II. Key Issues from the previous visit (if there was a visit before)

No.	Major findings that need to be improved	Action	Time line	Responsible person
2.1				
2.2				
2.3				
2.4				

HEW QUALITY REVIEW

III. HEW case management performance (quality of care) assessment of selected cases
(Select at least two most recent cases for each classification)

Classifications of the sick child 2 month to 5 years -including severe classification	#Classifications seen=A	Agreement between case management tasks		
		Assess and classify B	Classify and treat (DSD)*** C	Classify and Stated follow-up date D
		#Agree	#Agree	# Agree
Pneumonia				
Severe pneumonia or Very severe disease				
Malaria				
Very severe febrile diseases/Complicated measles				
Diarrhoea :No/some dehydration				
Severe dehydration/Dysentery/persistent/severe persistent diarrhoea				
Severe uncomplicated malnutrition				
Severe complicated malnutrition				
Total classifications seen in SC				
**Age below 2 months**				
Very severe disease				
Total classifications seen in SYI				

*SC= sick child (2-59 months); **SYI= sick young infant (up to 2 months); ***DSD= Correct Dose, Schedule and Duration.

Guide on how to fill the grid

A= Tally the total number of selected cases against each main classification of reviewed U5 children

B= Tally the number of classifications that agree with assessment among the reviewed U5 children.

C= Tally the number of classifications that agree with treatment among the reviewed U5 children

D= Tally the number of classifications that agree with the follow up date stated among the reviewed U5 children (when the sick U5 child has more than one health problems that need follow up, take the shortest follow up date that comes first for all the classifications)

III a. Children with severe classifications (as given by HEW) referred correctly

(From iCCM register review of at least 2 selected cases per each classification)

	Severe classifications that need referral (as given by HEW)	Total number	referred	Remarks
1	Severe Pneumonia or very severe disease			
2	Very severe febrile disease or and C. measles			
3	Diarrhea: severe dehydration/ dysentery/persistent or severe persistent diarrhoea			
4	Severe Complicated malnutrition/severe anemia/anemia			

III b. Children with non-severe classifications and compliance to follow up within the treatment period

(From iCCM register review of at least 2 selected cases per each classification)

	Non-severe classifications (as given by HEW)	Total number	Received follow up care	Remarks
1	Pneumonia			
2	Malaria			
3	Diarrhea			

III c. Treatment outcome for those non severe classification cases /cases that have received follow-up care/

	Classifications	Outcomes						Visit done before appointed FU date
		Total no.	# Same	# Improved	# Worsened	# Died	# Unknown*	
1	Pneumonia							
2	Malaria							
3	Diarrhea							

(From iCCM register review of at least two selected cases per each classification)

**these are sick U5 children who didn't receive follow-up care or outcome is not recorded*

III d. Children checked for well child care in the reporting period (cases selected for register review)

	**Age of sick child**	**Child care**	**Total number cases (from the two case per each selected classification)**	**Number checked**
1	≥ 6 months	Vitamin A status		
2	<24 months	Immunization status		
3	≥24 months	Deworming status		

DATA REVIEW

IV. **Number of children managed and reported by HEW in the last calendar month-review of report (Examine HMIS reporting form, *Compare with what is recorded in the register)***

Reporting period covered: (21.06/11_dd /mm/yyyy to 20/07/11_dd /mm/yyyy)

No	**Classification (including severe classification)**	**From Reporting form**	**Re-abstraction from Register**	**Difference**	**Remark**
1	Pneumonia cases				
2	Diarrhea cases				
3	Malaria cases				
4	New SAM cases				
5	Summation of classification (1-4)				
6	Total number of U5 children seen*				

***Current HIS does not capture number of under five children seen**

SUPPLY REVIEW

V. Logistics

Va. Essential Job aids in place (in use) on the day of visit (Put a √ mark)

	**Item**	**Yes**	**No**	**Remark**
1	Chart booklet			
2	IMNCI Registration books			
3	Family health card			
4	OTP card (where service is available)			

V b. Essential Functional Equipment's on the day of visit

	**Item**	**Yes**	**No**	**Remark**
1	Watch with second's arm			
2	Weighing scale - Baby lying or Salter scale with bowel			
3	MUAC tape			
4	Thermometer			
5	Newborn Ambu-bag			

Vc. Drugs and supplies-Check the expiry date for all the drugs and supplies available (Put a √ mark)

No	Oral drugs and supplies	Available on day of visit (√)		Out of stock in the last one month?-if yes write number of days			To anticipate stock out/monitor appropriate use of drugs and supplies			unaccounted quantity	Should be refilled by (date)
							Received	Used	Balance		
		Yes	No	Yes	# of days	No					
1	ORS Sachets										
2	Cotrimoxazole tablets										
3	Artemether Lumefentrine (Coartem) tablets										
4	Chloroquine syrup bottles										
5	RUTF (Plumpy Nut or BP100)* Sachets										
6	Amoxicillin for OTP* tablets										
7	Mebendazole/ Albendazole tablets										
8	Vitamin A capsules										
9	Zinc tablets										
10	Paracetamol tablets										
11	TTC eye ointment tubes										
12	Vitamin K ampoules										
13	2cc syringe and needle										
14	Examination gloves cartoon										
15	RDT reagent test kits										

*for HP that provides OTP service

V d: Drugs and supplies stored in appropriate manner

	Appropriate manner includes all of the following:	Yes	No	Remark
1	Storage is free from rodents or insects;			
2	Protected from sunlight			
3	Sufficient space for the quantity;			
4	Dry space and free from flooding			

VI. ORT corner *(Put √ mark)*

**Service**	**Yes**	**No**	Remark
ORT corner available (at least; a measuring jug,2cups, spoon, clean water, ORS)			
ORT corner functional (ORS solution given according to Plan B-registered)			

KNOWLEDGE OF HEWS

(Yes means they provided all answers as listed, they can use their job aids)

VII. Assessment of Knowledge –tell HEW to refer to job aids to answer the questions

			**HEW 1***		**HEW2***		**HEW3***	
	**Questions**	**Answers**	**Yes**	**No**	**Yes**	**No**	**Yes**	**No**
1	Cough and difficult breathing							
1.1	What is the correct breathing per minute cut off for the following?<br>Infant less than 2 month<br>Child 2-12 months<br>Child 12-59 months	(60/min or more						
		50/min or more						
		40/min or more						
1.2	Could you tell the correct doses of Co-trimoxazole for?<br>Sick child weighing 12 kg<br>Sick child age 8 months	Pediatrics tablet 3 tablets twice daily for five days<br>Pediatrics tablet 2 tablets twice daily for five days						
2	Diarrhoea		**Yes**	**No**	**Yes**	**No**	**Yes**	**No**
2.1	What are the 4 rules of home management for a child with diarrhea (plan A? (Do not prompt)	Give extra fluid (breastfeed more frequently for EBF infant)						
		Continue feeding						
		Give zinc						

			HEW 1*		HEW2*		HEW3*	
	**Questions**	**Answers**	**Yes**	**No**	**Yes**	**No**	**Yes**	**No**
		When to return						
2.2	Could you tell the correct amount of ORS for a child with some dehydration<br>Sick child weighing 10 kg<br>Sick child age 6 months	750 ml or 700-900ml over four hours in the HP<br>400- 700 over four hours in the HP						
3	Danger Signs –mark all that are mentioned without prompting		**Yes**	**No**	**Yes**	**No**	**Yes**	**No**
3.1	What are the General Danger Signs (GDS) in a sick child 2 months up to 5 years?	Lethargic or unconscious						
		Unable to drink or breastfeed						
		History Convulsions						
		Vomits every thing						
3.2	What are the signs of<br>possible serious Bacterial infection<br>(PSBI)/Severe disease in the sick young<br>infant birth to 2 months?	Not feeding well						
		Convulsions						
		Fast Breathing/Severe chest						
		in-drawing						
		Fever or low body						
		temperature						
		stimulated						
		No movement even when stimulated						

***Put NA if HEW was not assessed**

VIII. Mention the Essential Newborn Care actions (Put √ mark)

	Actions	HEW1*		HEW2*		HEW3*	
	**Answers**	**Yes**	**No**	**Yes**	**No**	**Yes**	**No**
1	Deliver baby on to mother's abdomen or into her arms						
2	Dry baby's body with dry towel; wipe eyes; wrap with another dry one and cover head						
3	Assess breathing, if not breathing or gasping or if breathing is <30 breaths per minute, then resuscitate.						
4	Tie the cord two finger from abdomen and another tie two fingers from the 1st one. Cut between the two ties and separate the baby from the placenta.						
5	Place the baby in skin-to skin contact with his mother and on the breast to initiate breastfeeding						
6	Apply Tetracycline eye ointment once to the newborn's eyes						
7	Give Vitamin K, 1mg IM on anterior mid-thigh						
8	Weigh baby properly						
	Advice mother to delay bathing of the baby for 24 hours after birth						
	Provide 4 postnatal visits during at 6-24 hour, 3rd day, 7th day and 6th week						

***Put NA if HEW was not assessed**

IX. Summarize the findings and secure agreement from the HEWs

IX a. Main Positive Findings (strengths):

1. ____

2. ____

3. ____

4. ____

IX b. Findings that need to be improved (weaknesses):

No.	Major findings that need to be improved	Action	Time line	Responsible person
1				
2				
3				
4				

Annex 7 SC and SYI compilation Forms

SC iCCM SYI iCCM complilation
complilationform Mayform May 3 2011.xlsx

Annex 8 Sample Planning Template for health post

iCCM quarterly planning form for health post

From___________to __________Zone_________Woreda_________PHCU/HC______HP______

		Month 1				Month 2				Month 3				Month 4			
Sn.	Major activities	W1	W2	W3	W4	W1	W2	W3	W4	W1	W2	W3	W4	W1	W2	W3	W4
1	**Create demand for iCCM**																
1.1	Announce regular working hours in the HP to kebele leaders, vCHW and community members through meetings, church gathering etc																
1.2	Conduct regular and continued orientation and sensitization of kebele leaders, community members, CBOs, CHP and others through meetings, church, religious gatherings, schools and others																
1.3	Conduct regular meetings with CHPs/health development army and discuss about the expected U5 children treated in each *gotte*																
1.4	Provide regular training to CHPs/HDAs about newborn and U5 child danger signs and exchange of immediate information by using FHC																
1.5	Use caretakers whose U5 children cured as a spontaneous promoters to attract others and build thrust																
2	**Service availability and quality**																
2.1	Start regular opening/function of HP to start implementation of iCCM and others services																

2.2	Use other opportunities like, home visits, outreach services, campaigns and community meetings to provide iCCM service																
2.3	Establish functional ORT corner and make sure always it is clean																
2.4	Provide quality of iCCM service and be friendly to caretakers																
2.5	Follow referred severe classification cases to HCs and check their treatment outcome																
3	**Drugs and supplies**																
3.1	Timely request and transportation of supplies before stock out happen																
3.2	Ensure the availability and functioning of essential supplies , job aids and equipment (Weight scale, thermometer, chart booklet, Cotrimoxzazole, Coartem, RDT kit etc)																
4	**Newborn and young infants**																
4.1	List pregnant women regularly																
4.2	Assist delivery and work with CHPs to get notification of delivery with 24 hours																
4.3	Provide two PNC visit within 2 days and 7 days of delivery																
4.4	Conduct active surveillance of young infant regardless of sick or not in collaboration of CHPs/HAD																
5	**Report**																
5.1	Compile and report iCCM HMIS data to PHCU and monitor the level of service utilization against expected case load																

Annex 9 Sample Planning Template for Woreda health Office

iCCM quarterly planning form for Woreda health Office

From__________to _________Zone_________Woreda_________PHCU/HC____________HP_________

		Month 1				Month 2				Month 3				Month 4			
Sn.	Major activities	W1	W2	W3	W4	W1	W2	W3	W4	W1	W2	W3	W4	W1	W2	W3	W4
1	**Orientation of ZHD and Create demand for iCCM**																
1.1	Assist HEWs to orient and sensitize kebele leaders , community members , CBOs , CHPs and others thorough organizing separate meetings for ICCM, church , mosque, school , and others																
1.2	Brief WorHO , Woreda administration , HCs staffs about ICCM and linkage between HPs and HCs																
1.3	Discuss and reach in to consensus on HEWs working hours in the HPs and inform HEWs , woreda administration, kebele leaders , CHPs and community members																
2	**Supervision**																
2.1	Prepare supervision plan for each HPs to visit every month and submit to HPs , HC and WorHO																
2.2	Conduct monthly regular supportive supervision and follow up visit																
2.3	Provide written feedbacks to all 5 HPs to share experience and able to																

	know where they are																
2.4	Conduct monthly performance review meeting and mentoring with HEWs in the HC																
3	**Drugs and supplies**																
3.1	Ensure the availability of essential supplies , job aids and equipment																
3.2	Facilitate to provide supplies which is going to stock out soon or already stock out																
4	**IMNCI**																
4.1	Strengthening IMNCI service in HC and improve referral linkage between HPs and HCs																
5	**Report**																
5.1	Regular collection, analysis and use of iCCM follow up and HMIS data from HPs to assess quality of iCCM and its utilization																
5.2	Documenting best iCCM practice and share to others																
5.3	Start compilation of report and send to WorHO including one copy of the prefilled checklist																

Appendix S2

Form C: ICCM Supportive Supervision/ Follow-up checklist

I. Identification

1.1	Date of visit (dd /mm/yyyy)
1.2	How many weeks ago was the last visit: ______weeks.
1.3	Region: _______ Zone: __________ Woreda: ____________ Kebele/H. Post: _______
1.4	Kebele's total population;___________; Total U5 children in the kebele______________
1.5	Name of supervising health centre:___________
1.6	Name of HEW in charge:______________________
1.7	Lead supervisor's name:___________________<br>Responsibility (e.g. HEP supervisor, iCCM coordinator, HEP focal person)___________________<br>Organization (e.g. MOH, Implementing Partner [specify name])__________________
1.8	Was Direct Case Observation made? Yes__ No __________<br>Total number of sick U5 observed: ____;<br>Number of Sick Children (2 -59 months)___ Number of Sick Young Infant (0 up to 2 months)__<br>(*if you get sick children during your visit do direct case observation using the appropriate recording form annexed at the end of this form*)
1.9	ICCM registration book reviewed: Yes _____No __.<br>Total number of sick U5 children reviewed _____;<br>Number of Sick Children (2 -59 months)____; Number of Sick Young Infant (0 up to 2 months)_**1**_
1.10	Total number of HEWs in Health Post: 2__Name of HEW supervised: 1.___________; 2)_______________; 3)______________________
1.11	How many Voluntary Community Health Workers in this kebele?_________<br>a. Which of the below activities do they perform:<br>i. Mobilizing families to seek iCCM services _________yes ___ no ___ not yet begun<br>ii. Health promotion activities _________yes ___ no ___not yet begun

II. Key Issues from the previous visit (if there was a visit before)

No.	Major findings that need to be improved	Action	Time line	Responsible person
2.1				
2.2				
2.3				

HEW QUALITY REVIEW

III. HEW case management performance (quality of care) assessment of selected cases

(Select at least two most recent cases for each classification)

**Classifications of the sick child 2 month to 5 years -including severe classification**	**#Classifications seen=A**	**Agreement between case management tasks**		
		**Assess and classify B**	**Classify and treat (DSD)*** C**	**Classify and Stated follow-up date D**
		**#Agree**	**#Agree**	**# Agree**
Pneumonia				
Severe pneumonia or Very severe disease				
Malaria				
Very severe febrile diseases/Complicated measles				
Diarrhoea :No/some dehydration				
Severe dehydration/ Dysentery/persistent/ severe persistent diarrhoea				
Severe uncomplicated malnutrition				
Severe complicated malnutrition				
Total classifications seen in SC				
**Age below 2 months**				
Very severe disease				
Total classifications seen in SYI				

*SC= sick child (2-59 months); **SYI= sick young infant (up to 2 months); ***DSD= Correct Dose, Schedule and Duration.

<u>Guide on how to fill the grid</u>

A= Tally the total number of selected cases against each main classification of reviewed U5 children

B= Tally the number of classifications that agree with assessment among the reviewed U5 children.

C= Tally the number of classifications that agree with treatment among the reviewed U5 children

D= Tally the number of classifications that agree with the follow up date stated among the reviewed U5 children (when the sick U5 child has more than one health problems that need follow up, take the shortest follow up date that comes first for all the classifications)

III a. Children with severe classifications (as given by HEW) referred correctly

(From iCCM register review of at least 2 selected cases per each classification)

	Severe classifications that need referral (as given by HEW)	Total number	referred	Remarks
1	Severe Pneumonia or very severe disease			
2	Very severe febrile disease or and C. measles			
3	Diarrhea: severe dehydration/ dysentery/persistent or severe persistent diarrhoea			
4	Severe Complicated malnutrition/severe anemia/anemia			

III b. Children with non-severe classifications and compliance to follow up within the treatment period

(From iCCM register review of at least 2 selected cases per each classification)

	Non-severe classifications (as given by HEW)	Total number	Received follow up care	Remarks
1	Pneumonia			
2	Malaria			
3	Diarrhea			

III c. Treatment outcome for those non severe classification cases /cases that have received follow-up care/

(From iCCM register review of at least two selected cases per each classification)

	**Classifications**	**Outcomes**						Visit done before appointed FU date
		**Total no.**	**# Same**	**# Improved**	**# Worsened**	**# Died**	**# Unknown***	
1	Pneumonia							
2	Malaria							
3	Diarrhea							

**these are sick U5 children who didn't receive follow-up care or outcome is not recorded*

III d. Children checked for well child care in the reporting period (cases selected for register review)

	**Age of sick child**	**Child care**	**Total number cases (from the two case per each selected classification)**	**Number checked**
1	≥ 6 months	Vitamin A status		
2	<24 months	Immunization status		
3	≥24 months	Deworming status		

DATA REVIEW

IV. **Number of children managed and reported by HEW in the last calendar month-review of report (Examine HMIS reporting form, *Compare with what is recorded in the register)***
Reporting period covered: (21.06/11_dd /mm/yyyy to 20/07/11_dd /mm/yyyy)

No	**Classification (including severe classification)**	**From Reporting form**	**Re-abstraction from Register**	**Difference**	**Remark**
1	Pneumonia cases				
2	Diarrhea cases				
3	Malaria cases				
4	New SAM cases				
5	Summation of classification (1-4)				
6	Total number of U5 children seen*				

***Current HIS does not capture number of under five children seen**

SUPPLY REVIEW

V. Logistics

Va. Essential Job aids in place (in use) on the day of visit (Put a √ mark)

	**Item**	**Yes**	**No**	**Remark**
1	Chart booklet			
2	IMNCI Registration book for Sick children 2-59 months of age			
3	IMNCI Registration book for Sick young infants 0-2 months of age			
4	Family health card			
5	OTP card (where service is available)			

V b. Essential Functional Equipment on the day of visit

	**Item**	**Yes**	**No**	**Remark**
1	Watch with second's arm			
2	Weighing scale - Baby lying or Salter scale with bowel			
3	MUAC tape			
4	Thermometer			
5	Newborn Ambu-bag			

Vc. Drugs and supplies-Check the expiry date for all the drugs and supplies available (Put a √ mark)

No	Oral drugs and supplies	Available on day of visit (√)		Out of stock in the last one month?-if yes write number of days			To anticipate stock out/monitor appropriate use of drugs and supplies			unaccounted quantity	Should be refilled by (date)
							Received	Used	Balance		
		Yes	No	Yes	# of days	No					
1	ORS Sachets										
2	Cotrimoxazole tablets										
3	Artemether Lumefantrine (Coartem) tablets										
4	Chloroquine syrup bottles										
5	RUTF (Plumpy Nut or BP100)* Sachets										
6	Amoxicillin for OTP* tablets										
7	Mebendazole/ Albendazole tablets										
8	Vitamin A capsules										
9	Zinc tablets										
10	Paracetamol tablets										
11	TTC eye ointment tubes										
12	Vitamin K ampoules										
13	2cc syringe and needle										
14	Examination gloves cartoon										
15	RDT reagent test kits										

*for HP that provides OTP service

V d: Drugs and supplies stored in appropriate manner

	Appropriate manner includes all of the following:	Yes	No	Remark
1	Storage is free from rodents or insects;			
2	Protected from sunlight			
3	Sufficient space for the quantity;			
4	Dry space and free from flooding			

VI. ORT corner *(Put √ mark)*

**Service**	**Yes**	**No**	Remark
ORT corner available (at least; a measuring jug,2cups, spoon, clean water, ORS)			
ORT corner functional (ORS solution given according to Plan B-registered)			

KNOWLEDGE OF HEWS

(Yes means they provided all answers as listed, they can use their job aids)

VII. Assessment of Knowledge –tell HEW to refer to job aids to answer the questions

			HEW 1*		HEW2*		HEW3*	
	**Questions**	**Answers**	**Yes**	**No**	**Yes**	**No**	**Yes**	**No**
1	Cough and difficult breathing							
1.1	What is the correct breathing per minute cut off for the following?<br>Infant less than 2 month<br>Child 2-12 months<br>Child 12-59 months	(60/min or more						
		50/min or more						
		40/min or more						
1.2	Could you tell the correct doses of Co-trimoxazole for?<br>Sick child weighing 12 kg<br>Sick child age 8 months	Pediatrics tablet 3 tablets twice daily for five days<br>Pediatrics tablet 2 tablets twice daily for five days						
2	Diarrhoea		**Yes**	**No**	**Yes**	**No**	**Yes**	**No**
2.1	What are the 4 rules of home management for a child with diarrhea (plan A? (Do not prompt)	Give extra fluid (breastfeed more frequently for EBF infant)						
		Continue feeding						
		Give zinc						
		When to return						
2.2	Could you tell the correct amount of ORS for a child with some dehydration<br>Sick child weighing 10 kg<br>Sick child age 6 months	750 ml or 700-900ml over four hours in the HP<br>400- 700 over four hours in the HP						
3	Danger Signs –mark all that are mentioned without prompting		**Yes**	**No**	**Yes**	**No**	**Yes**	**No**
3.1	What are the General Danger Signs (GDS) in a sick child 2 months up to 5 years?	Lethargic or unconscious						
		Unable to drink or breastfeed						
		History Convulsions						
		Vomits every thing						
3.2	What are the signs of possible serious Bacterial infection (PSBI)/Severe disease in the sick young infant birth to 2 months?	Not feeding well						
		Convulsions						
		Fast Breathing/Severe chest						
		in-drawing						
		Fever or low body						
		temperature						
		stimulated						
		No movement even when stimulated						

***Put NA if HEW was not assessed**

VIII. Mention the Essential Newborn Care actions (Put √ mark)

	Actions	HEW1*		HEW2*		HEW3*	
	**Answers**	**Yes**	**No**	**Yes**	**No**	**Yes**	**No**
1	Deliver baby on to mother's abdomen or into her arms						
2	Dry baby's body with dry towel; wipe eyes; wrap with another dry one and cover head						
3	Assess breathing, if not breathing or gasping or if breathing is <30 breaths per minute, then resuscitate.						
4	Tie the cord two finger from abdomen and another tie two fingers from the 1st one. Cut between the two ties and separate the baby from the placenta.						
5	Place the baby in skin-to skin contact with his mother and on the breast to initiate breastfeeding						
6	Apply Tetracycline eye ointment once to the newborn's eyes						
7	Give Vitamin K, 1mg IM on anterior mid-thigh						
8	Weigh baby properly						
	Advice mother to delay bathing of the baby for 24 hours after birth						
	Provide 4 postnatal visits during at 6-24 hour, 3rd day, 7th day and 6th week						

***Put NA if HEW was not assessed**

IX. Summarize the findings and secure agreement from the HEWs

IX a. Main Positive Findings (strengths):

1. ____

2. ____

3. ____

4. ____

IX b. Findings that need to be improved (weaknesses):

No.	Major findings that need to be improved	Action	Time line	Responsible person
1				
2				
3				
4				

IXc. Further suggestions if any:

MANAGEMENT OF THE SICK CHILD AGE 2 MONTHS UP TO 5 YEAR

Name: ______________________________Age: ______Sex_______ Weight: _______ Temperature: _____°C

ASK: What are the child's problems? ____ Initial visit? __Follow-up Visit? ___

ASSESS (Circle all signs present)		CLASSIFY
CHECK FOR GENERAL DANGER SIGNS		
NOT ABLE TO DRINK OR BREASTFEED<br>VOMITS EVERYTHING<br>CONVULSIONS	LETHARGIC OR UNCONSCIOUS<br>CONVULSING NOW	General danger signs present Yes__ No__
DOES THE CHILD HAVE COUGH OR DIFFICULT BREATHING? Yes___ No___		
For how long? ___ Days	Count the breaths in one minute. ____ breaths per minute. Fast breathing?<br>Look for chest in-drawing.<br>Look and listen for stridor.	
DOES THE CHILD HAVE DIARRHEA? Yes ____ No ___		
For how long? ___Days<br>Is there blood in the stool?	-Look at the child's general condition. Is the child:<br>Lethargic or unconscious?<br>Restless or irritable?<br>-Look for sunken eyes.<br>-Offer the child fluid. Is the child:<br>Not able to drink or drinking poorly?<br>Drinking eagerly, thirsty?<br>-Pinch the skin of the abdomen. Does it go back:<br>Very slowly (longer than 2 seconds)?<br>(slowly less than 2 seconds)	
DOES THE CHILD HAVE FEVER? (by history/feels hot/temperature > 37.5℃ or above) Yes___ No___		
Malaria Risk: High Low No<br>if low or no malaria risk, then ask:<br>Has the child travelled outside this area during the last one month?<br>If yes, has he been to a malarious area?<br>For how long has the child had fever? __ Days<br>If more than 7 days, has the fever been present every day?<br>Has child had measles within the last three months?	-Look or feel for stiff neck.<br>-Look for runny nose<br>-Look for signs of MEASLES:<br>-Generalized rash and<br>-One of these: cough, runny nose, or red eyes.<br>*-Do RDT: Positive__Negative__ Not done_*	
If the child has measles now<br>or within the last 3 months:	Look for mouth ulcers.<br>Look for pus draining from the eye.<br>Look for clouding of the cornea.	
DOES THE CHILD HAVE AN EAR PROBLEM? Yes___ No___		
Is there ear pain?<br>Is there ear discharge? If Yes, for how long? ____ Days	Look for pus draining from the ear.	
THEN CHECK THE SICK CHILD BELOW 6 MONTHS OF AGE FOR MALNUTRITION	-Look For visible severe wasting<br>-Look for pitting oedema of both feet.	
THEN CHECK FOR MALNUTRITION THE SICK CHILD AGE 6 MONTHS AND ABOVE	-Measure MUAC<br>MUAC Less than 11cm<br>MUAC 11 cm to <12 cm<br>MUAC ≥12 cm and above<br>-Check for Pitting oedema of both feet<br>-Complication: Pneumonia, watery diarrhoea/dysentery, fever<br>-If MUAC <11cm or oedema of both feet and no medical complication do appetite test: fail/ pass	
THEN CHECK FOR ANEMIA	Look for palmar pallor: Severe pallor? Some pallor?	
CHECK FOR POSSIBLE SYMPTOMATIC HIV INFECTION Ask: what is the HIV status of the mother Positive__, Negative__, Unknown___<br>What is the HIV status of the child: PCR: Positive__, Negative__, Unknown___<br>Antibody: Positive__, Negative__, Unknown___		
CHECK THE CHILD'S IMMUNIZATION (age<2 year) AND VITAMIN A STATUS Circle immunizations/vitamin A needed today.<br>_______ BCG ______ Pentavalent-1 _______ Pentavalent-2 ________ Pentavalent-3<br>____________ PCV-1 __________ PCV-2 ____________ PCV-3<br>_______ OPV 0 ________ OPV 1 _______ OPV 2 _____ OPV 3 _______ Measles ________ VITAMIN A _______________ Mebendazole / Albendazole		RETURN FOR NEXT IMMUNIZATION/ VITAMIN A ON:<br>______________<br>(DATE)
ASSESS CHILD'S FEEDING if child has ANEMIA OR MODERATE ACUTE MALNUTRITION or is less than 2 years old.		FEEDING PROBLEMS:
Do you breastfeed your child? Yes____ No ____<br>If Yes, how many times in 24 hours? ___times. Do you breastfeed during the night? Yes___ No___.<br>Do you empty one breast before you shift to the other one?<br>Does the child take any other food or fluids even water? Yes___ No ___<br>If Yes, what food or fluids?<br>How many times per day? ___times. What do you use to feed the child?<br>If the child has moderate acute malnutrition: How large are servings?<br>Does the child receive his own serving? ___ Who feeds the child and how?<br>During this illness, has the child's feeding changed? Yes ____ No ____, if Yes, how? ______________________________		
ASSESS FOR OTHER PROBLEMS	COUNSEL THE MOTHER ABOUT HER OWN HEALTH	

MANAGEMENT OF THE SICK YOUNG INFANT AGE BIRTH UP TO 2 MONTHS

Name: __________________________________ Age: ______ Sex:________ Weight: _______ kg Temperature: ____°C

ASK: What are the infant's problems? _________________________________ Initial visit? ___ Follow-up Visit? ___

ASSESS (Circle all signs present) CLASSIFY

ASSSESS FOR BIRTH ASPHYXIA (immediately after birth)
- Not breathing
- Is breathing poorly (less than 30 per minute)
- Gasping

ASSESS FOR BIRTH WEIGHT AND GESTATIONAL AGE (the first 7 days of life)
Ask gestational age; <32wks, 32-<37wks, ≥ 37wks
Weigh the baby: <1500g, 1500-<2500g, ≥2500g

CHECK FOR VERY SEVERE DISEASE and LOCAL INFECTION

Has the infant had convulsions?

Has the infant stopped feeding well?

-Count the breaths in one minute. ____breaths per minute
Repeat if 60 or more ________ Fast breathing?
-Look for severe chest in-drawing.
-Look at umbilicus. Is it red or draining pus?
-Fever (temperature ≥ 37.5°C or feels hot) or body temperature below 35.5°C (or feels cool)
-Look for skin pustules.
-Look at young infant's movements.
- Does the infant move only when stimulated?
- Does the infant not move even when stimulated?

CHECK FOR JAUNDICE
-Look for jaundice?
Are the palms and soles yellow?
Are, skin on the face or eyes yellow?
Is age less than 24 hours or more than 14 days

DOES THE YOUNG INFANT HAVE DIARRHOEA? Yes _____ No ______

For how long? _______ Days
Is there blood in the stools?

-Look at the young infant's general condition:
Does the infant move only when stimulated?
Does the infant not move even when stimulated?
Is the infant restless or irritable?
-Look for sunken eyes.
-Pinch the skin of the abdomen. Does it go back:
Very slowly (longer than 2 seconds)? Slowly?

CHECK FOR HIV INFECTION
Ask: what is the HIV status of the mother Positive____, Negative_____ , Unknown______
What is the HIV status of the child Positive____, Negative_____, Unknown______

THEN CHECK FOR FEEDING PROBLEM OR LOW WEIGHT

Is the infant breastfed? Yes _____ No _____
If Yes, how many times in 24 hours? _____ times
Do you empty one breast before switching to the other? Yes ___No__
Do you increase frequency and length of breastfeeding during illness? Yes __No__
Does the infant receive any other foods or drinks, even water? Yes ___ No ____
If Yes, ask for any reason and how often?
if yes what do you use to feed the child?

Determine weight for age. Low ___ Not Low ___

If the infant is feeding less than 8 times in 24 hours, is taking any other food or drinks, or is under weight for age AND has no indications to refer urgently to hospital:

ASSESS BREASTFEEDING:
Has the infant breastfed in the previous hour?
- If infant has not fed in the previous hour, ask the mother to put her infant to the breast. Observe the breastfeed for 4 minutes.
- If the infant was fed during the last hour, ask the mother if she can wait and tell you when the infant is willing to feed again
Is the infant positioned well? To check positioning, look for:
- Infant's head and body straight Yes ___No ___
- Facing the breast nose against nipple Yes ___No ___
- Infant's body close to mother's body Yes ___No ___
- Mother supporting the whole body Yes ___No ___

- the infant able to attach? To check attachment, look for:
- Chin touching breast Yes __No __
- Mouth wide open Yes __No __
- Lower lip turned outward Yes __No __
- More areola above than below the mouth Yes __No __

no attachment at all *not well attached* *good attachment*

-Is the infant suckling effectively (that is, slow deep sucks, sometimes pausing)?

not suckling at all *not suckling effectively* *suckling effectively*

-Clear blocked nose if it interferes with breastfeeding
Look for ulcers or white patches in the mouth (thrush).

CHECK THE YOUNG INFANT'S IMMUNIZATION STATUS Circle immunizations needed today.

______ BCG	______ Pentavalent-1	______ PCV-1
______ OPV 0	______ OPV 1	

Return for next immunization on: ______________ (Date)

ASSESS OTHER PROBLEMS: COUNSEL THE MOTHER ABOUT HER OWN HEALTH
